# Supplementary material for: The Effect of Bee Venom and Melittin on Glioblastoma Cells in Zebrafish Model
Source: Molecules. 2025 Aug 7;30(15):3306. doi: 10.3390/molecules30153306 (PMC12348512; doi:10.3390/molecules30153306)
Supplement: Supplementary file 1 [file molecules-30-03306-s001.zip › molecules-3761014-supplementary.pdf]

Data File C:\Chem32\...12062021_22_23\bee venom 12062021 22 2021-06-12 10-04-05\081-0104.D
Sample Name: bee venom 22

=====================================================================

Acq. Operator : SYSTEM  Seq. Line : 4
Acq. Instrument : Biotex  Location : 81
Injection Date : 12-Jun-21 11:31:15 AM  Inj : 4
Inj Volume : 20.000 µl
Method : C:\Chem32\1\Data\bee_venom_12062021_22_23\bee venom 12062021 22 2021-06-12 10-04-05\Bee Vinom.M (Sequence Method)
Last changed : 12-Jun-21 10:04:05 AM by SYSTEM

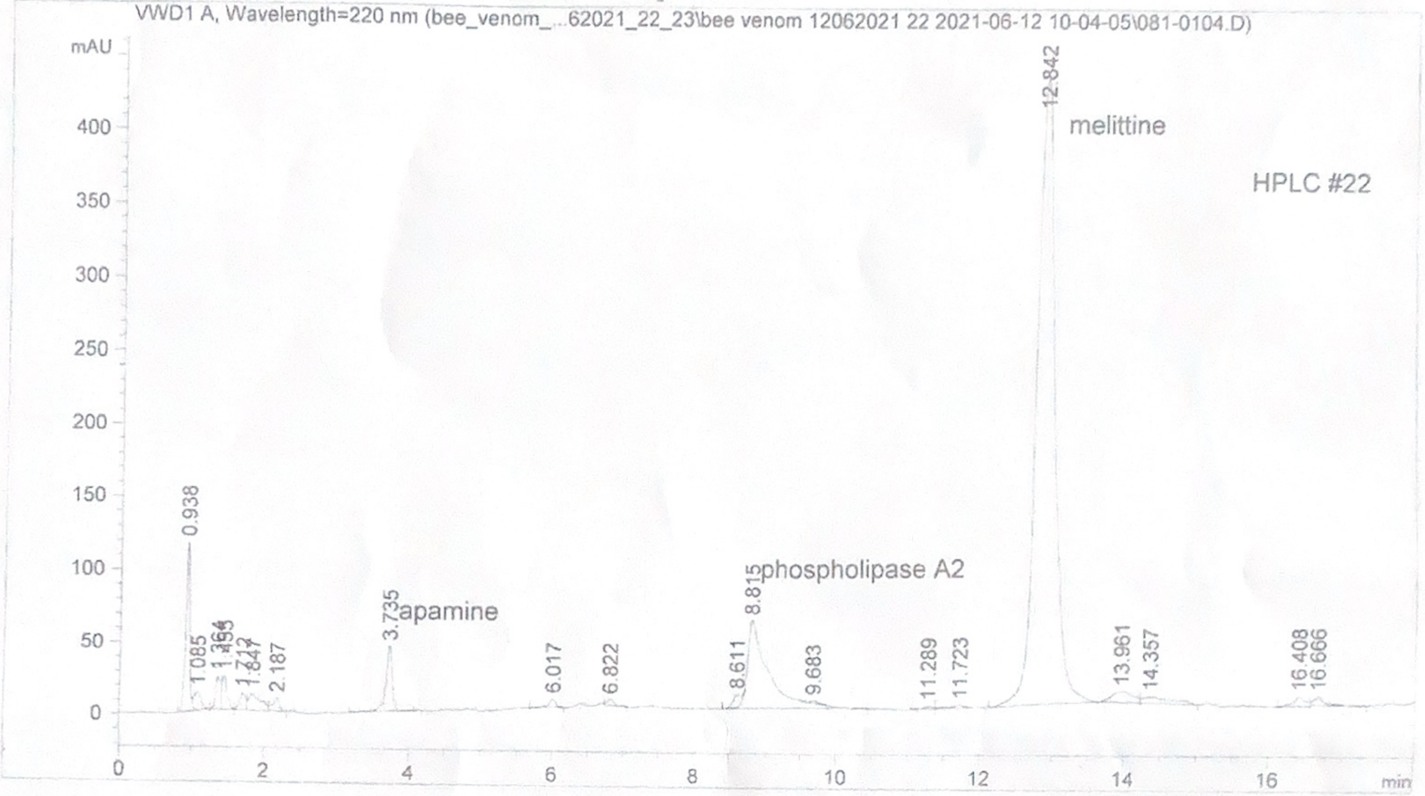


=====================================================================

Area Percent Report

=====================================================================

Sorted By : Signal
Multiplier : 1.0000
Dilution : 1.0000
Use Multiplier & Dilution Factor with ISTDs

Signal 1: VWD1 A, Wavelength=220 nm

| Peak # | RetTime [min] | Type | Width [min] | Area [mAU*s] | Height [mAU] | Area % |
|---|---|---|---|---|---|---|
| 1 | 0.938 | BV R | 0.0588 | 457.89664 | 116.91374 | 4.2873 |
| 2 | 1.085 | VB E | 0.0976 | 87.20738 | 13.17927 | 0.8165 |
| 3 | 1.364 | BV | 0.0630 | 103.89165 | 22.93762 | 0.9728 |
| 4 | 1.455 | VB | 0.0695 | 115.87114 | 23.38507 | 1.0849 |
| 5 | 1.712 | BV | 0.0747 | 60.86367 | 11.80803 | 0.5699 |
| 6 | 1.847 | VV | 0.1568 | 135.74600 | 11.24561 | 1.2710 |
| 7 | 2.187 | VB | 0.0962 | 61.07169 | 9.05307 | 0.5718 |
| 8 | 3.735 | BV R | 0.0920 | 280.25375 | 45.68183 | 2.6241 |
| 9 | 6.017 | VB | 0.1267 | 54.10600 | 5.85528 | 0.5066 |
| 10 | 6.822 | VB | 0.0999 | 28.63952 | 4.28307 | 0.2682 |
| 11 | 8.611 | BV E | 0.1164 | 68.13902 | 8.67730 | 0.6380 |
| 12 | 8.815 | VV R | 0.2711 | 1268.09766 | 61.50299 | 11.8734 |
| 13 | 9.683 | VB E | 0.1285 | 16.02966 | 1.80305 | 0.1501 |

| Peak # | RetTime [min] | Type | Width [min] | Area [mAU*s] | Height [mAU] | Area % |
|---|---|---|---|---|---|---|
| 14 | 11.289 | BV | 0.1236 | 19.58802 | 2.18413 | 0.1834 |
| 15 | 11.723 | VB | 0.3091 | 46.24734 | 1.96800 | 0.4330 |
| 16 | 12.842 | BB | 0.2647 | 7448.39355 | 427.00275 | 69.7403 |
| 17 | 13.961 | BV | 0.3136 | 153.35844 | 7.47406 | 1.4359 |
| 18 | 14.357 | VB | 0.4005 | 133.80496 | 4.46541 | 1.2528 |
| 19 | 16.408 | BV | 0.1555 | 68.39114 | 6.03507 | 0.6404 |
| 20 | 16.666 | VB | 0.1661 | 72.58269 | 5.99166 | 0.6796 |

Totals : 1.06802e4 791.44700

*** End of Report ***

Bee Venom Lab LLC

Bee Venom Lab LLC

6, Mitskevichi str. Apt #48. Tbilisi 0194. Georgia

Phone:(+99532)2383824; Email: info@beevenomlab.com

Website: www.beevenomlab.com

## Complete list and CAS of components of Bee Venom

| Component | CAS number | % |
|---|---|---|
| Melittin | 20449-79-0 | 40-75 |
| Apamin | 24345-16-2 | 2-3 |
| MCD-Peptide (Peptide401) | 32908-73-9 | 2-3 |
| Adolapin | 79029-92-8 | 1.0 |
| Protease Inhibitor | 66701-25-5 | <0.8 |
| Tertiapin | 910044-56-3 | 0.1 |
| Melittin F | 37231-28-0 | 0.01 |
| Procamine A, B | 51943-80-7 | 1.4 |
| Cardiopep | 54511-11-4 | <0.7 |
| Hyaluronidase | 9001-54-1 | 1.5-2.0 |
| Phospholiase A2 | 9001-84-7 | 10-12 |
| Histamine | 56-92-8 | 0.6-1.6 |
| Norepinephrine | 51-41-2 | 0.1 -0.7 |
| 6 Phospholipids | 123465-35-0 | 4-5 |
| γ-Aminobutyric | 56-12-2 | <0.5 |
| Secarpin | 58694-50-1 | <0.1 |
| **Minerals:** | | |
| Phosphorus | 7723-14-0 | 3.1 |
| Magnesium | 7439-95-4 | <0.1 |
| Calcium | 7440-70-2 | <0.1 |
| Copper | 7440-50-8 | <0.1 |

Sincerely,

Ketevan Gurasashvili

CEO
Bee Venom Lab LLC

BEE VENOM LAB LLC
405185525
